# Statement of retraction: insulin-loaded PLGA microspheres for glucose-responsive release

**DOI:** 10.1080/10717544.2025.2604934

**Published:** 2025-12-19

We, the authors, Editor and Publisher of the Drug Delivery, have retracted the following article:

Wu, J. Z., Williams, G. R., Li, H. Y., Wang, D. X., Li, S. D., & Zhu, L. M. (2017). Insulin-loaded PLGA microspheres for glucose-responsive release. *Drug Delivery*, *24*(1), 1513–1525. https://doi.org/10.1080/10717544.2017.1381200

After publication, concerns were raised by a third party about the integrity of the data in Figure 5 of the article. Additional image integrity concerns were identified by the publisher, specifically regarding Figure 1D and 2E.

When approached for an explanation, the authors responded, however, concerns remain. As verifying the validity of published work is core to the integrity of the scholarly record, we are therefore, retracting the article.

We have been informed in our decision-making by our editorial policies and the COPE guidelines.

The retracted article will remain online to maintain the scholarly record, but it will be digitally.

Watermarked on each page as ‘Retracted’.

